# Epidemiology and Clinical Characteristics of Human Coronaviruses-Associated Infections in Children: A Multi-Center Study

**DOI:** 10.3389/fped.2022.877759

**Published:** 2022-04-12

**Authors:** Kyo Jin Jo, Soo-Han Choi, Chi Eun Oh, HyeonA Kim, Bong Seok Choi, Dae Sun Jo, Su Eun Park

**Affiliations:** ^1^Department of Pediatrics, Pusan National University Children's Hospital, Yangsan, South Korea; ^2^Department of Pediatrics, Pusan National University Hospital, Busan, South Korea; ^3^Department of Pediatrics, Kosin University College of Medicine, Busan, South Korea; ^4^Department of Pediatrics, School of Medicine, Kyungpook National University, Daegu, South Korea; ^5^Department of Pediatrics, Jeonbuk National University Medical School, Jeonju, South Korea

**Keywords:** human coronavirus, multiplex polymerase chain reaction, children, multi-center study, prognosis

## Abstract

**Background:**

Human coronaviruses (HCoVs) are associated with upper respiratory tract infections. Although studies have analyzed the clinical and epidemiological characteristics of HCoV-associated infections, no multi-center studies have been conducted in Korean children. We aimed to describe the epidemiology and clinical characteristics of HCoV-associated infection in children.

**Methods:**

We retrospectively reviewed medical records of children in whom HCoVs were detected using multiplex reverse transcriptase-polymerase chain reaction amplification in five centers from January 2015 to December 2019.

**Results:**

Overall, 1,096 patients were enrolled. Among them, 654 (59.7%) patients were male. The median age was 1 year [interquartile range (IQR), 0–2 years]. HCoVs were identified mainly in winter (55.9%). HCoV-229E, HCoV-OC43, and HCoOV-NL63 were detected mainly in winter (70.9, 55.8, and 57.4%, respectively), but HCoV-HKU1 was mainly identified in spring (69.7%). HCoV-OC43 (66.0%) was detected most frequently, followed by HCoV-NL63 (33.3%), and HCoV-229E (7.7%). Two different types of HCoVs were co-detected in 18 samples, namely. Alphacoronavirus-betacoronavirus co-infection (*n* = 13) and, alphacoronavirus-alphacoronavirus co-infection (*n* = 5). No betacoronavirus-betacoronavirus co-infection was detected. Patients were diagnosed with upper respiratory tract infection (41.4%), pneumonia (16.6%), acute bronchiolitis (15.5%), non-specific febrile illness (13.1%), croup (7.3%), and acute gastroenteritis (5.1%). There were 832 (75.9%) hospitalized patients with a median duration of hospitalization of 4 days (IQR, 3–5 days); 108 (9.9%) patients needed supplemental oxygen with 37 (3.4%) needing high-flow nasal cannula or mechanical ventilation. There were no deaths.

**Conclusion:**

HCoV-associated infections exhibit marked seasonality with peaks in winter. Patients with lower respiratory tract infection, a history of prematurity, or underlying chronic diseases may progress to a severe course and may need oxygen therapy.

## Introduction

Human coronaviruses (HCoVs) were identified as human respiratory pathogens in the 1960's (1). In addition to the recently discovered severe acute respiratory syndrome coronavirus 2 (SARS-CoV-2) in 2019, other HCoVs, such as severe acute respiratory syndrome coronavirus, Middle East respiratory syndrome coronavirus, SARS-CoV-2 and four common HCoVs (i.e., HCoV-229E, HCoV-OC43, HCoV-NL63, and HCoV-HKU1) are known to cause respiratory infections in humans (2, 3).

Common HCoVs are found worldwide and cause diseases predominantly during winter and spring in temperate climates (4, 5). They are most frequently associated with upper respiratory tract infections (URIs) and are characterized by rhinorrhea, nasal congestion, sore throat, sneezing, and cough that may be associated with mild fever (4, 6–8), as well as acute otitis media or asthma exacerbations (4, 7–9). HCoVs are sometimes also associated with lower respiratory tract infections, including croup, bronchiolitis, and pneumonia, primarily in infants and immunocompromised patients (7, 10–16).

In Korea, while some studies analyzed clinical or epidemiological characteristics of HCoV-associated infection (14, 17–19), they were either single-center studies or focused on patients of all ages. Till date, there have been no multi-center studies focusing on children in Korea; hence, this study aimed to investigate the epidemiologic and clinical characteristics of HCoV-associated infections in children admitted to various hospitals in the Republic of Korea.

## Materials and Methods

### Participants

We conducted a retrospective chart review from January 2015 to December 2019 for children (0 to 18 years old) in whom HCoVs were detected using multiplex reverse transcriptase-polymerase chain reaction amplification (RT-PCR) at five hospitals in the southern part of Korea: Pusan National University Children's Hospital, Pusan National University Hospital, Kosin University Gospel Hospital, Kyungpook National University Children's Hospital, and Jeonbuk National University Children's Hospital. The following clinical characteristics of patients were analyzed: age, sex, date of admission, admission to intensive care unit, clinical diagnosis, history of prematurity and underlying chronic diseases, need for oxygen therapy, high-flow nasal cannula oxygen therapy or mechanical ventilation, presence of fever, presence of respiratory or gastrointestinal symptoms, presence of infiltrates on chest radiographs, length of hospital stay, and results of virologic study. We excluded patients in whom HCoVs were detected using RT-PCR 48 h or more after hospital admission. Patients who were diagnosed with Kawasaki disease, urinary tract infection, or other viral and bacterial infections were also excluded. To investigate the seasonal fluctuations of each HCoVs, the year was divided into spring (March-May), summer (June-August), autumn (September-November), and winter (December-February). The present study protocol was reviewed and approved by the Institutional Review Board of the Pusan National University Yangsan Hospital (approval No. 05-2020201). The requirement for informed consent was waived due to the retrospective study design.

### Laboratory Testing

Specimens of nasopharyngeal swabs for RT-PCR analyses were collected and two different RT-PCR tests were performed. The first test was a respiratory virus multiplex RT-PCR (Seoul Clinical Laboratories, Gyeonggi-do, Republic of Korea), and it was performed at all five hospitals. This test commenced in January 2015 at all the five hospitals. Testing was done for HCoV-229E, HCoV-OC43, HCoV-NL63, human adenovirus, human metapneumovirus, influenza A virus, influenza B virus, parainfluenza virus types 1-3, respiratory syncytial virus (RSV) A, RSV B, human rhinovirus A, human rhinovirus B, human rhinovirus C, and human bocavirus. The second test was the FILMARRAY Respiratory Panel (BioFiredx, Salt Lake City, USA) and the study was performed at two of the five hospitals. One hospital started in September 2018, and the other in January 2019. This test assessed for HCoV-229E, HCoV-OC43, HCoV-NL63, HCoV-HKU1, human adenovirus, human metapneumovirus, human rhinovirus, influenza A virus, influenza B virus, parainfluenza virus types 1- 4, RSV, *Bordetella pertussis, Chlamydophila pneumoniae*, and *Mycoplasma pneumoniae*.

### Statistical Analyses

The Wilcoxon signed-rank test (non-parametric) and the χ^2^ test were used to compare non-normally distributed continuous variables and continuous variables, respectively. Statistical analyses were performed using SPSS version 25.0 (SPSS Inc., Chicago, IL, USA). A value of *P* < 0.05 was considered significant.

## Results

Nasopharyngeal swab samples were obtained from 238,637 children and respiratory viruses were detected in 22,542 (9.4%) children. HCoVs were detected in 1,406 of 22,542 (6.2%) patients; 310 patients were excluded. In total, 1,096 patients were enrolled during the study period.

**Figure 1:**
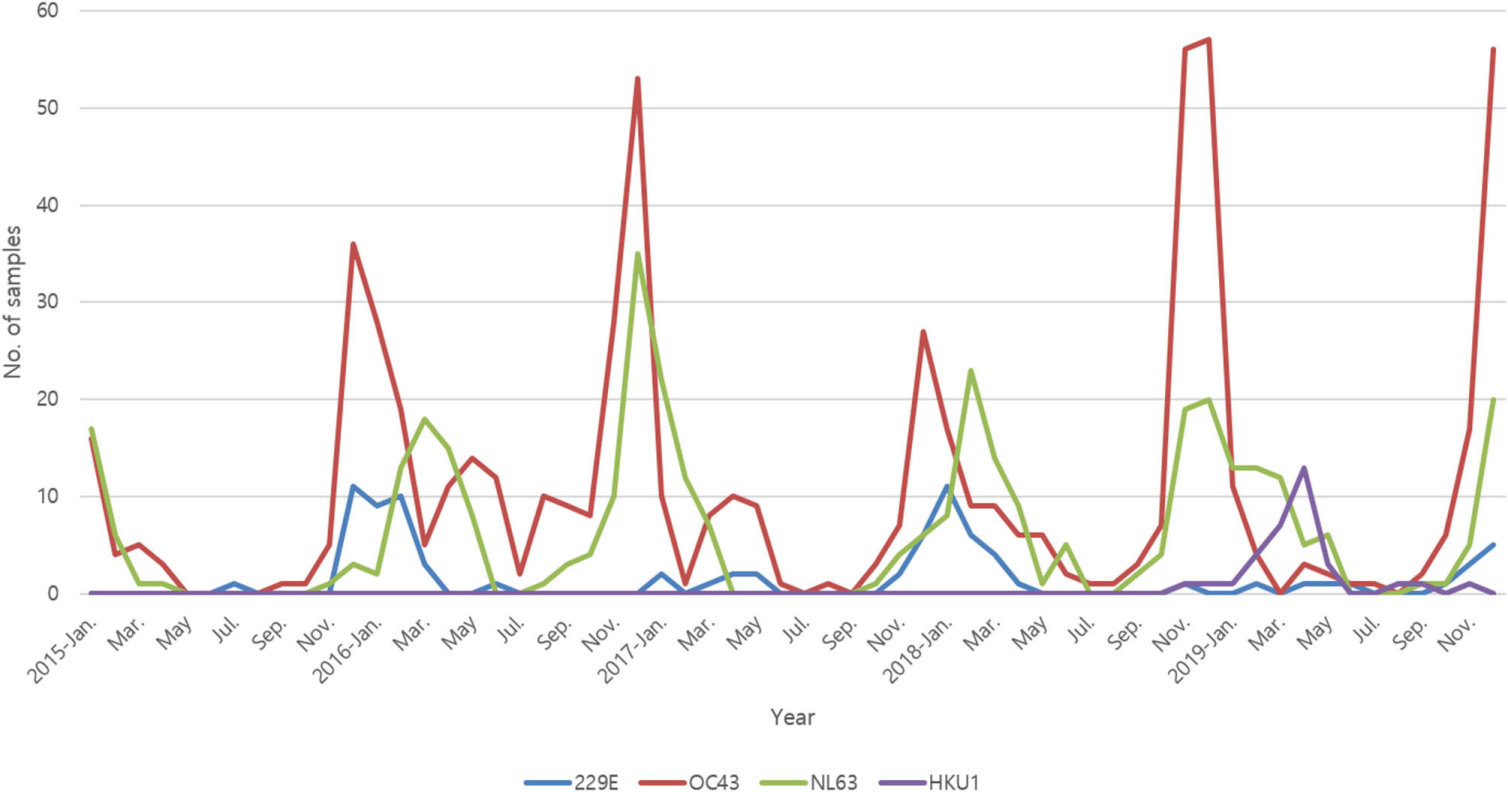
Monthly distribution of HCoVs between January 2015 and December 2019.

Among them, 664 (60.6%) patients were male, and the median age was 1 year [interquartile range (IQR), 0–2 years]. Co-infection with two different types of HCoVs was observed in 18 patients. The HCoVs were identified mainly in winter (55.9%) (see Figure 1). HCoV-OC43 (66.0%) was the most commonly detected HCoV, followed by HCoV-NL63 (33.3%) and HCoV-229E (7.7%). In the case of HCoV-HKU1, FILMARRAY Respiratory Panel (BioFiredx, Salt Lake City, USA) was performed in two institutions for about 1 year, and 18.1% of the samples that detected HCoVs using FILMARRAY Respiratory Panel (BioFiredx, Salt Lake City, USA) were confirmed to be positive for HCoV-HKU1.

HCoV-229E, HCoV-OC43, and HCoV-NL63 were mainly detected in winter (70.9, 55.8, and 57.4%, respectively), but HCoV-HKU1 was mainly detected in spring (69.7%). HCoV-229E and HCoV-OC43 are alphacoronavirus, and HCoV-NL63 and HCoV-HKU1 are betacoronavirus. Two different types of HCoVs were co-detected in 18 samples, which included. Alphacoronavirus-betacoronavirus co-infection (*n* = 13) and alphacoronavirus-alphacoronavirus co-infection (*n* = 5). No betacoronavirus-betacoronavirus co-infection was detected. Other viruses were co-detected with HCoVs in 44.5% of patients. Of these, two and three non-HCoV respiratory viruses were co-detected in 78 and 11 patients, respectively. Among them, RSV (28.9%) was the most detected virus, followed by human rhinovirus (27.7%) and human adenovirus (19.2%) (Table 1). The patients were diagnosed with URI (41.4%), pneumonia (16.6%), acute bronchiolitis (15.5%), non-specific febrile illness (13.1%), croup (7.3%), and acute gastroenteritis (5.1%). Of the total, 270 (24.6%) patients had a history of prematurity or underlying chronic diseases.

**Table 1:** Frequency of viral detections from January 2015 to December 2019.

	**No. of samples positive for each virus**										
	**HCoV-229E**	**HCoV-OC43**	**HCoV-NL63**	**HCoV-HKU1**	**RSV**	**RV**	**HAdV**	**Flu**	**PIV**	**HBoV**	**HMPV**
HCoV-229E (*n* = 86)	-	5	2	0	9	10	12	14	0	2	2
HCoV-OC43 (*n* = 624)	5	-	11	0	126	103	69	25	25	19	7
HCoV-NL63 (*n* = 371)	2	11	-	0	35	46	29	17	13	11	10
HCoV-HKU1 (*n* = 33)	0	0	0	-	1	6	5	0	1	0	0
All HCoVs (*n* = 1,096^*^)	-	-	-	-	170^†^	163^†^	113^†^	53^†^	39	32	18^†^

* *Two different types of HCoVs were co-detected in 18 samples*.

† *Due to the samples with ≥2 non-HCoV co-detections, the sum of the number of samples positive for each non-HCoV is greater than the number of samples with any non-HCoV co-detection. RSV, respiratory syncytial virus; RV, Rhinovirus; HAdV, human adenovirus; Flu, influenza; PIV, parainfluenza virus; HBoV, human bocavirus; HMPV, human metapneumovirus*.

Of the 1,096 patients, 832 (75.9%) were hospitalized, and the median duration of hospitalization was 4 days (IQR, 3–5 days). Oxygen supply was needed in 108 (9.9 %) patients, and 37 (3.4%) needed high-flow nasal cannula oxygen therapy or mechanical ventilation. These 37 patients were diagnosed with pneumonia (51.4%), acute bronchiolitis (16.2%), croup (16.2%), URI (13.5%), and asthma attack (2.7%). Out of them, 23 (62.2%) had a history of prematurity or underlying chronic diseases. Among the hospitalized patients, respiratory virus co-detection was found in 18 (48.6%) patients. Human rhinovirus (36.5%) was the most commonly detected virus, followed by RSV (27.3%), influenza viruses (13.6%), human metapneumovirus (13.6%), and parainfluenza virus (4.5%). When comparing patients who needed high flow-nasal cannula oxygen therapy or mechanical ventilation with those who did not, no significant differences in patient's age and sex were found (*P* = 0.349, *P* = 0.620) (Table 2). Patients in need of high-flow nasal cannula oxygen therapy or mechanical ventilation had significantly more history of prematurity or underlying chronic diseases (*P* < 0.001), and the ratio of pneumonia was high (*P* < 0.001).

**Table 2:** Clinical characteristics associated with HCoV infection: comparison of patients who needed high-flow nasal cannula oxygen therapy or mechanical ventilation with those who did not.

**Clinical feature**	Severe group^*^ **(*N* = 37)**	**Not severe group** ** (*N* = 1,059)**	***P*-Value**
**Age (years), median (IQR)**	0.0 (0.0–4.0)	1.0 (0.0–2.0)	0.620
**Male (%)**	26 (68.4)	640 (59.5)	0.349
**HCoV subtype**			0.124
HCoV-229E (%)	0 (0.0)	79 (7.5)	
HCoV-OC43 (%)	20 (54.1)	588 (55.5)	
HCoV-NL63 (%)	16 (43.2)	342 (32.3)	
HCoV-HKU1 (%)	0 (0.0)	33 (3.1)	
2 HCoVs co-detections	1 (2.7)	17 (1.6)	
**Diagnosis**			
Upper respiratory infection (%)	5 (13.5)	449 (42.4)	0.001
Pneumonia (%)	19 (51.4)	163 (15.4)	<0.001
Acute bronchiolitis (%)	6 (16.2)	164 (15.5)	1.000
Non-specific febrile illness (%)	0 (0.0)	144 (13.6)	0.028
Croup (%)	6 (16.2)	74 (7.0)	0.082
Acute gastroenteritis (%)	0 (0.0)	56 (5.3)	0.279
Asthma attack (%)	1 (2.7)	4 (0.4)	0.416
Encephalitis (%)	0 (0.0)	5 (0.5)	1.000
**Viral co-infection (%)**	18 (48.6)	484 (45.7)	0.592
**History of prematurity or underlying chronic diseases (%)**	23 (62.2)	247 (23.3)	<0.001

* *Severe group includes patients who need high-flow nasal cannula oxygen therapy or mechanical ventilation. IQR, interquartile range*.

According to the type of HCoV, there were no significant differences in patient's sex (*P* = 0.173) and patients who were detected with HCoV-229E were older than those detected with HCoV-OC43 (*P* = 0.001) (Table 3). The most common clinical diagnosis associated with all four types of HCoVs was URI. While, HCoV-229E and HCoV-HKU1 were more frequently associated with non-specific febrile illness (*P* = 0.027), HCoV-OC43 and HCoV-NL63 were more frequently associated with pneumonia (*P* = 0.001) and croup (*P* < 0.001), respectively. There were significant differences in the viral co-infection in HCoV-229E- and HCoV-NL63-infected patients (*P* < 0.001). There were no significant differences in the history of prematurity or underlying chronic diseases (*P* = 0.539).

**Table 3:** Clinical characteristics associated with each type of HCoV detection.

**Clinical feature**	**HCoV-229E** ** (*N* = 86)**	**HCoV-OC43 ** ** (*N* = 624)**	**HCoV-NL63** ** (*N* = 371)**	**HCoV-HKU1** ** (*N* = 33)**	***P*-Value**
**Age (years), median (IQR)**	1.5 (0.0–5.0)	1.0 (0.0–2.0)	1.0 (0.0–3.0)	1.0 (0.0–3.0)	0.001
**Male (%)**	57 (66.3)	372 (59.6)	222 (59.8)	15 (45.5)	0.227
**Diagnosis**					
Upper respiratory infection (%)	38 (44.2)	263 (42.1)	145 (39.1)	17 (51.5)	0.455
Pneumonia (%)	10 (11.6)	126 (20.2)	51 (13.8)	0 (0.0)	0.001
Acute bronchiolitis (%)	10 (11.6)	104 (16.7)	52 (14.0)	4 (12.1)	0.470
Non-specific febrile illness (%)	18 (20.9)	78 (12.5)	42 (11.3)	8 (24.2)	0.027
Croup (%)	3 (3.5)	14 (2.2)	62 (16.7)	2 (6.1)	<0.001
Acute gastroenteritis (%)	5 (5.8)	34 (5.5)	16 (4.3)	2 (6.1)	0.774
Asthma attack (%)	1 (1.2)	2 (0.3)	2 (0.5)	0 (0.0)	0.525
Encephalitis (%)	1 (1.2)	3 (0.5)	1 (0.3)	0 (0.0)	0.434
**Viral co-infection (%)**	44 (52.2)	305 (48.9)	134 (36.1)	12 (36.4)	<0.001
**History of prematurity or underlying chronic diseases (%)**	20 (23.3)	148 (23.7)	102 (27.5)	7 (21.2)	0.539

*IQR, interquartile range*.

Comparing single infections and viral co-infections, there were no significant differences in patient's age and sex (*P* = 0.148, *P* = 0.931) (Table 4). The clinical diagnosis was also significantly different between both groups, as pneumonia and acute bronchiolitis were more frequent in viral co-infections (*P* < 0.001, *P* < 0.001), whereas non-specific febrile illness was more prevalent in HCoV single infections (*P* < 0.001). There was no significant difference in the requirement for hospital admission (*P* = 0.116); however, the duration of hospital admission was longer in the viral co-infection group than in the single-infection group (*P* < 0.001). There were no significant differences in the history of prematurity or underlying chronic diseases and clinical severity.

**Table 4:** Clinical characteristics associated with HCoV infection: comparison between single infection and viral co-infection.

**Clinical feature**	**Total HCoV infection** ** (*N* = 1,096)**	**Single HCoV infections** ** (*N* = 608)**	**HCoV Co-infections** ** (*N* = 488)**	***P-*Value**
**Age (years), median (IQR)**	1.0 (0.0–2.0)	1.0 (0.0–2.0)	1.0 (0.0–3.0)	0.148
**Male (%)**	654 (59.7%)	364 (59.9%)	290 (59.4%)	0.931
**Diagnosis**				
Upper respiratory infection (%)	454 (41.4)	262 (43.1)	192 (39.4)	0.234
Pneumonia (%)	182 (16.6)	78 (12.8)	104 (21.3)	<0.001
Acute bronchiolitis (%)	170 (15.5)	71 (11.7)	99 (20.3)	<0.001
Non-specific febrile illness (%)	144 (13.1)	102 (16.8)	42 (8.6)	<0.001
Croup (%)	80 (7.3)	53 (8.7)	27 (5.5)	0.058
Acute gastroenteritis (%)	56 (5.1)	38 (6.2)	18 (3.7)	0.076
Asthma attack (%)	5 (0.5)	1 (0.2)	4 (0.8)	0.178
Encephalitis (%)	5 (0.5)	3 (0.5)	2 (0.4)	1.000
**History of prematurity or underlying chronic diseases (%)**	270 (24.6)	163 (26.8)	107 (21.9)	0.073
Needed hospital admission (%)	832 (75.9)	450 (74.0)	382 (78.3)	0.116
**Days of hospital stay, median (IQR)**	4.0 (3.0–5.0)	3.0 (3.0–5.0)	4.0 (3.0–6.0)	<0.001
**Needed O**_**2**_ **supply (%)**	108 (9.9)	62 (10.2)	46 (9.4)	0.746
**Needed HFNC or mechanical ventilation (%)**	37 (3.4)	19 (3.1)	18 (3.7)	0.730

*IQR, interquartile range; HFNC, high-flow nasal cannula oxygen therapy*.

## Discussion

We investigated the prevalence pattern of HCoVs for 5 consecutive years, from 2015 to 2019. During the study period, HCoV-229E, HCoV-OC43, and HCoV-NL63 showed marked seasonality with peaks in winter and were rarely detected in summer. Meanwhile, HCoV-HKU1 showed a marked seasonality with peaks in spring. HCoV-OC43 was the most commonly detected HCoV. These findings are consistent with those of earlier studies (4, 14, 16, 18, 20). However, our finding that HCoV-HKU1 showed a marked seasonality with peaks in spring is not consistent with that of earlier studies, which have shown that HCoV-HKU1 infections peak in winter and spring (20, 21). We could only investigate HCoV-HKU1 in two centers and for only a short duration. Therefore, further studies are required to better understand the patterns of activity of HCoV-HKU1.

In our study, two different types of HCoVs were co-detected in 18 samples. They were alphacoronavirus-betacoronavirus co-infection (*n* = 13) and alphacoronavirus-alphacoronavirus co-infection (*n* = 5). Betacoronavirus-betacoronavirus co-infection was not detected in any samples. The lack of significant sequence identity between the alphacoronavirus spike proteins and betacoronavirus cousins (<25%) suggests that they would be unlikely to have significant cross-reactivity. SARS-CoV-2 is also a betacoronavirus, and interest in cross-reactive antibodies against seasonal betacoronaviruses and SARS-CoV-2 have increased since the latter occurred in 2019. There were some studies about cross-reactive antibodies against seasonal betacoronaviruses and SARS-CoV-2 (22–26). Although it was reported that seasonal betacoronavirus antibodies led to a rise in the level of cross-reactive antibodies against SARS-CoV-2, its association with protection is not yet clear (26). Further studies would be needed to determine this interaction, though due to the high level of sequence and structural homology of betacoronaviruses spike proteins, such a cross-reactivity.

In our study, there were no cases of death despite some patients having had a history of prematurity or underlying chronic diseases. Most patients (75.9%) were hospitalized, and 9.9% of patients needed oxygen supply or high-flow nasal cannula oxygen therapy; however, all patients recovered and were discharged. There are a few studies about the clinical features of HCoV-associated infection in adults (14–16). One study retrospectively investigated HCoV-associated infections in patients of all ages from January 2018 to March 2020 (14). In that study, there were no cases of deaths in children, and the mortality rate was high in older individuals. The authors suggested that the reasons for the high mortality rate in older patients with infection can be the presence of comorbid diseases, the poor immunity, or the high probability of pneumonia. In our study, 24.6% of patients had a history of prematurity or underlying chronic diseases, and 16.6% of patients were diagnosed with pneumonia, but there were no deaths. Our study showed that unlike adults, HCoV-associated infection generally has a mild to moderate evolution with a favorable clinical outcome in children.

HCoV-229E, HCoV-OC43, HCoV-NL63, and HCoV-HKU1 are known to be associated with URI in children (4, 6, 8). Our study also showed that URI was the most common clinical diagnosis associated with all four types of HCoVs. There are a few studies showing that HCoV-OC43, and HCoV-NL63 are associated with lower respiratory tract infections (17, 27, 28). Our study showed HCoV-OC43 was more frequently associated with pneumonia. Compared with other HCoVs, HCoV-NL63 is more frequently associated with croup, and it is the second most common cause after parainfluenza virus type 1 (29, 30). Our study also showed that HCoV-NL63 was more frequently associated with croup than the other HCoVs. In our study, five patients were diagnosed with encephalitis. Among them, three patients were detected with HCoV-OC43, and the other two patients were detected with HCoV-229E and HCoV-NL63. Coronavirus can cause neurological diseases in animals; however, the exact association with neurological diseases in humans is not yet known. There are few studies about the association between HCoVs and neurological diseases. A Finnish study provided evidence of HCoV-OC43 infection in six patients with acute neurologic episodes (31). Another case study reported the presence of acute demyelinating encephalitis accompanying HCoV-OC43 respiratory tract infection, wherein HCoV-OC43 RNA was detected in the spinal fluid using PCR (32). However, further investigations will be needed to establish whether HCoVs are related causally to any neurologic disease in humans.

In our study, the 3.4% of patients who needed high flow nasal cannula oxygen therapy or mechanical ventilation were detected with HCoV-NL63 or HCoV-OC43. Among them, HCoV-NL63 and HCoV-OC43 were detected in 44.7 and 55.3% of patients, respectively. In a Belgian study, among children aged ≤ 5 years admitted to the hospital, the highest burden was associated with HCoV-OC43, followed by HCoV-NL63 (16). In adults, a study showed that patients detected with HCoV-OC43 were twice as likely to require oxygen or intubation than those with other strains (15); another study showed that mortality was higher in patients infected with HCoV-OC43 than those infected with other HCoVs (14). Comparing single infections and viral co-infections in our study, there were no significant differences in the requirement for hospital admission and clinical severity, as characterized by the need for oxygen supply, high-flow nasal cannula oxygen therapy, or mechanical ventilation. Considering these points, it is thought that viral co-infection is not related to the severity of the disease. Our study showed HCoV-OC43 was more frequently associated with pneumonia and that HCoV-NL63 was more frequently associated with croup. Because pneumonia and croup are diseases that can cause difficulty in breathing, we surmise that patients with HCoV-NL63 and HCoV-OC43 needed more high-flow nasal cannula oxygen therapy or mechanical ventilation. The reason for the virulent prognosis of HCoVs is still unclear, and further investigation is needed to better understand this topic.

Our study has some limitations that should be noted. First, the study was retrospective in nature, which has inherent bias. Second, respiratory multiplex RT-PCR testing was not performed on all patients who visited the hospitals. If the symptoms were mild or typical of URI, it was highly likely that the test was not performed; and if hospitalization was required, the test would have, in all likelihood, been performed. The proportion of patients who were diagnosed with non-specific febrile illness and URI may have been higher than that reported in our study. Third, we only investigated HCoV-HKU1 in two centers and for a short duration. Further studies with longer duration are needed to better understand the epidemiology and clinical features of HCoV-HKU1.

HCoV-associated infection has marked seasonality with peaks in winter, and it generally has a mild-to-moderate evolution with a favorable clinical outcome. Patients with lower respiratory tract infection, a history of prematurity, or underlying chronic diseases may need oxygen and may progress to a severe course, necessitating careful observation.

## Data Availability Statement

The original contributions presented in the study are included in the article/Supplementary Material, further inquiries can be directed to the corresponding author.

## Author Contributions

KJ helped to write the initial draft and collect the data. S-HC helped to collect the data and performed statistical analysis. CO helped to collect the data contributed on revisions of the initial draft. HK, BC, and DJ helped to collect the data. SP conceived the study and contributed on revisions of the initial draft. All authors approved the final version of the report.

## Funding

This work was supported by a 2-year Research Grant from Pusan National University.

## Conflict of Interest

The authors declare that the research was conducted in the absence of any commercial or financial relationships that could be construed as a potential conflict of interest.

## Publisher's Note

All claims expressed in this article are solely those of the authors and do not necessarily represent those of their affiliated organizations, or those of the publisher, the editors and the reviewers. Any product that may be evaluated in this article, or claim that may be made by its manufacturer, is not guaranteed or endorsed by the publisher.
