# Supplementary material for: Epidemiology and Clinical Characteristics of Human Coronaviruses-Associated Infections in Children: A Multi-Center Study
Source: Front Pediatr. 2022 Apr 12;10:877759. doi: 10.3389/fped.2022.877759 (PMC9039334; doi:10.3389/fped.2022.877759)
Supplement: Supplementary file 1 [file Table_1.DOCX]

**Supplementary Table 1**┃Characteristics of 37 cases who needed high flow nasal cannula oxygen therapy or mechanical ventilation.

| **Age** | **Sex** | **Diagnosis** | **Symptom** | **Co-morbidity** | **HCoV subtype** | **Combined pathogen** |
| --- | --- | --- | --- | --- | --- | --- |
| 0 | M | Pneumonia | Fever, dyspnea | Epilepsy, preterm infant | NL63 | None |
| 0 | M | Acute bronchiolitis | Cough | None | NL63 | None |
| 0 | M | Croup | Cough, stridor, dyspnea | None | NL63 | None |
| 0 | M | Acute bronchiolitis | Fever, cough | Allergic dis. | NL63 | None |
| 0 | M | Croup | Cough, stridor, dyspnea | Congenital heart dis. | NL63 | HRV |
| 0 | M | Croup | Fever, cough, dyspnea | None | NL63 | HRV |
| 0 | M | Acute bronchiolitis | Fever, cough, dyspnea | None | NL63 | HRV |
| 0 | M | Pneumonia | Fever, cough, dyspnea | Congenital heart dis. | OC43 | None |
| 0 | M | Acute bronchiolitis | Fever, chough, chest retraction | None | OC43 | None |
| 0 | M | Pneumonia | Cough, coryza | Allergic dis. | OC43 | None |
| 0 | M | Acute bronchiolitis | Cough, dyspnea | None | OC43 | RSV, HRV |
| 0 | M | Pneumonia | Fever, cough, dyspnea | Laryngomalacia | OC43 | RSV |
| 0 | F | Croup | Fever, cough, dyspnea | None | NL63 | None |
| 0 | F | Pneumonia | Fever, dyspnea | Congenital heart dis., laryngomalacia | NL63 | HMPV |
| 0 | F | URI | Cough | Congenital heart dis., preterm infant | OC43 | None |
| 0 | F | URI | Cough | Congenital heart dis. | OC43 | None |
| 0 | F | URI | Coryza, dyspnea | Congenital heart dis. | OC43 | None |
| 0 | F | URI | Fever, cough, dyspnea | Congenital heart dis. | OC43 | IFV |
| 0 | F | Pneumonia | Fever, cough, dyspnea | None | OC43 | RSV |
| 0 | F | Pneumonia | Fever, cough, dyspnea | Congenital heart dis. | OC43 | RSV, HRV |
| 1 | M | Pneumonia | Fever, cough, chest retraction | Hematologic dis. | OC43 | None |
| 1 | M | Croup | Fever, cough, dyspnea | None | NL63 | RSV |
| 1 | M | Pneumonia | Fever, cough, dyspnea | None | OC43 | PIV |
| 1 | M | Pneumonia | Cough, chest retraction | None | OC43 | HRV |
| 1 | F | Pneumonia | Fever, dyspnea | Epilepsy, muscular dystrophy | NL63 | None |
| 2 | M | URI | Fever, cough | None | OC43 | RSV, HAdV |
| 3 | M | Pneumonia | Cough, dyspnea | None | OC43 | HRV |
| 4 | M | Pneumonia | Fever, dyspnea | Muscular dystrophy | NL63 | None |
| 4 | F | Pneumonia | Fever, chough, chest retraction | Epilepsy, muscular dystrophy | NL63 | None |
| 4 | F | Pneumonia | Fever, chough, chest retraction | Epilepsy, hydrocephalus, allergic dis. | OC43 | None |
| 4 | F | Acute bronchiolitis | Fever, chough | Epilepsy, muscular dystrophy | OC43 | HMPV |
| 6 | M | Croup | Fever, cough, dyspnea | None | NL63 | HMPV |
| 6 | M | Asthma attack | Fever, cough, dyspnea | Epilepsy | OC43 | None |
| 6 | F | Pneumonia | Fever, cough | Hydrocephalus | NL63 | HRV, IFV |
| 7 | M | Pneumonia | Fever, cough, dyspnea | Immunodeficiency dis. | NL63, OC43 | IFV |
| 10 | F | Pneumonia | Cough, dyspnea | Muscular dystrophy | OC43 | None |
| 14 | F | Pneumonia | Fever, dyspnea | Epilepsy | NL63 | None |

M, male; F, female; Dis., disease; URI, upper respiratory infection; HRV, human rhinovirus; RSV, respiratory syncytial virus; HMPV, human metapneumovirus; IFV, influenza virus; PIV, parainfluenza virus; HAdV, human adenovirus.
